# Embryonic stem cell microenvironment enhances proliferation of human retinal pigment epithelium cells by activating the PI3K signaling pathway

**DOI:** 10.1186/s13287-020-01923-0

**Published:** 2020-09-23

**Authors:** Jiahui Liu, Liu Yang, Xiaoran Wang, Shoubi Wang, Zheqian Huang, Chaoyang Li, Ying Liu, Yaqi Cheng, Chengxiu Liu, Zhichong Wang

**Affiliations:** 1 Affiliated Dongguan People’s Hospital, Southern Medical University, Dongguan, China; 2 State Key Laboratory of Ophthalmology, Zhongshan Ophthalmic Center, Sun Yat-sen University, Guangzhou, 510060 China; 3 Affiliated Hospital of Qingdao University Medical College, Qingdao, China

**Keywords:** Embryonic stem cell microenvironment, Proliferation, PI3K pathway, Regenerative medicine

## Abstract

**Background:**

Retinal pigment epithelium (RPE) replacement has been proposed as an efficacious treatment for age-related macular degeneration (AMD), which is the primary cause of vision loss in the elderly worldwide. The embryonic stem cell (ESC) microenvironment has been demonstrated to enable mature cells to gain a powerful proliferative ability and even enhance the stem/progenitor phenotype via activation of the phosphoinositide 3-kinase (PI3K) signaling pathway. As the PI3K signaling pathway plays a pivotal role in proliferation and homeostasis of RPE, we hypothesize that the stemness and proliferative capability of RPE can be enhanced by the ESC microenvironment via activation of the PI3K signaling pathway.

**Methods:**

To investigate whether the ESC microenvironment improves the stem cell phenotype and proliferation properties of human RPE (hRPE) cells by regulating the PI3K signaling pathway, primary hRPE cells were cocultured with either ESCs or human corneal epithelial cells (CECs) for 72 h, after which their proliferation, apoptosis, cell cycle progression, and colony formation were assayed to evaluate changes in their biological characteristics. Gene expression was detected by real-time PCR and protein levels were determined by western blotting or immunofluorescence. LY294002, an antagonist of the PI3K signaling pathway, was used to further confirm the mechanism involved.

**Results:**

In comparison to hRPE cells cultured alone, hRPE cells cocultured with ESCs had an increased proliferative capacity, reduced apoptotic rate, and higher colony-forming efficiency. The expression of the stem cell-associated marker KLF4 and the differentiation marker CRALBP increased and decreased, respectively, in hRPE cells isolated from the ESC coculture. Furthermore, PI3K pathway-related genes were significantly upregulated in hRPE cells after exposure to ESCs. LY294002 reversed the pro-proliferative effect of ESCs on hRPE cells. In contrast, CECs did not share the ability of ESCs to influence the biological behavior and gene expression of hRPE cells.

**Conclusions:**

Our findings indicate that the ESC microenvironment enhances stemness and proliferation of hRPE cells, partially via activation of the PI3K signaling pathway. This study may have a significant impact and clinical implication on cell therapy in regenerative medicine, specifically for age-related macular degeneration.

## Background

Age-related macular degeneration (AMD) is one of the leading irreversible causes of vision loss in the elderly worldwide. With the increasing longevity of the population, the prevalence of AMD is rising annually [1–5]. Although the exact cause of AMD remains unclear, it is universally acknowledged that aged or dysfunctional retinal pigment epithelium (RPE) cells play a vital role in the initial pathogenesis of AMD [6]. Consequently, RPE replacement has been proposed as a treatment for AMD.

In 1991, an autologous pedicle RPE graft by Peyman et al. in a patient with end-stage AMD resulted in improved visual acuity, suggesting that RPE replacement could be an efficacious treatment [7]. Unfortunately, patients who require cell transplantation usually do not have enough viable RPE cells to repopulate the entire macula adequately [8]. In addition, lipofuscin and melanosomes undergoing significant age-related changes will influence phototoxicity and detract from the normal functioning of the RPE and hinder the benefits of RPE transplantation [9]. Researchers have successfully attempted to promote RPE cell proliferation and functional recovery by mesenchymal stem cells [10–12], such as umbilical cord Wharton’s jelly-derived mesenchymal stem cells (WJ-MSCs) [13]. But the inevitable immune rejection by allogeneic cells cannot be ignored. Recent studies in regenerative medicine have revealed that human embryonic stem cells (hESCs) and induced pluripotent stem cells (iPSCs) can be induced to differentiate into functional RPE cells, providing hope of using pluripotent stem cells to rescue visual function. However, certain problems with these approaches limited their clinical application, such as being costly and time-consuming, and having low efficiency and the potential for tumorigenesis [10, 14–16]. Consequently, the development of a convenient, efficient, and safe method to obtain sufficient viable autologous RPE cells is urgently required in AMD therapy.

Salero et al. discovered that RPE stem cells (RPESCs) are present in the human RPE layer, thus providing new insight for RPE replacement therapy [17]. RPESCs have a multipotency and self-renewal potential and can differentiate into RPE cells that produce neural and mesenchymal progeny. It was demonstrated by Blenkinsop et al. that RPESC-derived RPEs preserve the native RPE morphology, electrophysiology, and gene and protein expression patterns [18]. When grown on transwell polyester membranes, the RPESC-derived RPEs can be transplanted into rabbit subretinally and survive with retained characteristics in the subretinal space for at least 4 weeks [19]. Research has shown that proper culture conditions could turn off suppressive factors to activate RPESC proliferation [20]. Therefore, activating the surviving RPE within a patient’s eye is a strategy for replenishing the RPE layer and may also benefit the neural retina by producing favorable growth factors or improving RPE support of neural retinal cell function. Moreover, surgical injury and immunosuppression could potentially be avoided by this strategy. Therefore, it is crucial to explore ways to promote the proliferation and differentiation potential of RPESCs both in vitro and in vivo*.*

The embryonic stem cell (ESC) microenvironment can ameliorate or even reverse the aging process and enable mature cells to gain a powerful proliferative ability [21–25]. Yousef et al. have confirmed that the proliferation of mouse and human muscle progenitors can be enhanced by hESC-conditioned medium [24]. Similarly, our previous work demonstrated that the ESC microenvironment markedly improved the stem cell phenotype and proliferation properties of corneal epithelial cells and even human corneal endothelial cells that are considered unable to proliferate and that the PI3K signaling pathway is indispensable for these pro-regenerative effects [26–30]. Therefore, we hypothesize that the stemness and proliferative capability of RPE can be enhanced by the ESC microenvironment via activation of the PI3K signaling pathway. To test this hypothesis, the present study examined the effects of coculturing primary human RPE (hRPE) cells with ESCs on the proliferation, apoptosis, cell cycle progression, and colony formation of hRPE cells. Our findings may open a novel therapeutic avenue for AMD and other diseases of RPE dysfunction.

## Methods

### Cell culture

hRPE cells were extracted from eyecups of bulbus oculi subsequent to the application of corneal transplantation, performed at the State Key Laboratory of Ophthalmology (Guangzhou, China). The cell culture, storage, and subculture were performed following previously described guidelines [17]. hRPE cells were cultured in DMEM/F12 (Corning) with 10% FBS and 1% penicillin-streptomycin. Subsequent experiments were performed on the fourth or fifth generation of hRPE cells, when the cells had reached the optimum level of cell viability and morphology, in which the cell confluence rate reached between 70 and 90%. Cell identification was conducted using immunofluorescent staining of CRALBP protein, RPE65, and S-100 antibodies as described below.

Mouse ESCs were gifts from Professor Andy Peng Xiang. ESCs were cultured in knockout Dulbecco’s modified Eagle’s medium (DMEM; Gibco) with 10% FBS (Gibco), 0.1 mM non-essential amino acid (Gibco), 1% GlutaMAX media (Gibco), 0.055 mM 2-mercaptoethanol (Gibco), 5 × 10^5^ units leukemia inhibitory factor (Millipore, USA), and 1% penicillin-streptomycin. Green fluorescent protein-labeled ESCs were constructed as described previously [31] and grown in ESC culture medium.

The CEC cell line, established in our laboratory previously [32], used as a kind of differentiated cell, without indifferentiability, to coculture with RPE cells was cultured in DMEM with 10% FBS, 10 ng/ml human epidermal growth factor (hEGF, Pepro Tech, USA), 5 mg/ml insulin (Sigma, USA), 5 mg/ml human transferrin (Sigma), 0.4 mg/ml hydrocortisone (MB-Chem, India), 2 mM L-glutamine (Gibco), and 1% penicillin-streptomycin.

hRPE cells and CECs were stained with cell-labeling solution (CM- DiI or DiD, Invitrogen) according to the manufacturer’s protocol. 6 × 10^5^ DiD-labeled hRPE cells were plated in a 75-cm^2^ culture flask with 6 × 10^5^ green fluorescent protein-labeled ESCs or CECs in hRPE culture medium. hRPE cells were collected after 72 h using fluorescence-activated cell sorting (BD FACSAria Fusion, USA). For control groups, hRPE cells were cultured alone in hRPE culture medium.

### Cell cycle analysis

Cells were resuspended with 75% alcohol at − 20 °C overnight. After that, cells were incubated with 1 mg/ml RNase A stock solution for 30 min at 37 °C, stained with 100 μg/ml propidium iodide (BD) for 5 min at room temperature, and assessed on an LSRFortessa flow cytometer (BD). Data analysis was conducted using Modfit software.

### Apoptosis assay

We followed a previous method to evaluate the apoptosis assay [30]. Staining cells were assessed by BD LSRFortessa flow cytometer using Annexin V-APC/7-aminoactinomycin D (Invitrogen) according to the manufacturer’s protocol.

### Clone formation assay

The clone formation assay was performed as previously described [30]. Cells were seeded into 6-well plates (1000 hRPE cells/well) and cultured for 10 days. Clones were visualized by crystal violet staining and counted.

### CCK-8 cell proliferation assay

As previously described [30], hRPE cells (700 cells/well) were seeded in a 96-well plate. Twenty-four hours after seeding, CCK-8 reagent (Dojindo Molecular Technologies, Japan) was added to the cell culture media for 1 h at 37 °C. The cell proliferation curve was generated according to the optical density measured at 450 nm (BioTek, USA).

### RT-qPCR

Total RNA was isolated from cell cultures and tissues using a RNeasy Plus Mini kit (Qiagen, Germany) according to the manufacturer’s instructions and then quantified by absorption at 260 nm as previously described [31]. cDNA was synthesized with a PrimeScript™ RT Master Mix (Takara, Japan) and used for qPCR with SYBR® Premix Ex Taq™ (Takara) in a StepOnePlus thermal cycler (ABI, USA). The GAPDH gene was used as an internal control. The PCR primer sequences are listed in Table 1.

**Table 1:** Primer sequences

	Forward	Reverse
Human-GAPDH	CGTATTGGGCGCCTGGTCAC	ATGATGACCCTTTTGGCTCC
Human-cyclin A2	CGCTGGCGGTACTGAAGTC	GAGGAACGGTGACATGCTCAT
Human-KLF4	TCTCAAGGCACACCTGCGAA	TAGTGCCTGGTCAGTTCATC
Human-p21	TGTCCGTCAGAACCCATGC	AAAGTCGAAGTTCCATCGCTC
Human-p27	TAATTGGGGCTCCGGCTAACT	TGCAGGTCGCTTCCTTATTCC
Human-PAR2	TTGTGTTTGTGGTGGGTTTGC	ACCAGATGACAGAGAGGAGGT
Human-PDK2	CTTCAGCAAGTTCTCCCCGTC	TCGGGAAGCAGGTTGATCTCT
Human-PDK1	AGAGGGTTACGGGACAGATGC	GTCTTTGGGTTCTCTGCTGGG
Human-AKT	GGAGAGGAAGAGATGGATGCCT	CCACTTGCCTTCTCTCGAACC
Human-PI3K	AACACCGACCTCACAGTTTTT	CTCAAGCCACACATTCCACAG
Human-cyclin B1	GGTTGTTGCAGGAGACCATGT	AACATGGCAGTGACACCAACC
Human-cyclin D1	TTCATTTCCAATCCGCCCTCC	TGTGAGGCGGTAGTAGGACAG
Human- FAK	GTTATCCCAGTCCGAGGTCCA	TGACCTGGATAGATGCTGCCA
Human- CRALBP	AGCTGCTCAGAGGCTATGTGA	CCAGGGTAGCCAGCTTCAATG
Human- PEDF	TTCAAAGTCCCCGTGAACAAG	GAGAGCCCGGTGAATGATGG

### Western blot analysis

Protein expression in hRPE cells from four groups was assessed using western blotting as previously described [30]. The primary antibodies used are described in Table 2. The secondary antibody used was HRP-conjugated goat anti-rabbit IgG (1:2000, Sigma). Localization of antibodies was detected by an enhanced chemiluminescence kit (Amersham, Piscataway, NJ).

**Table 2:** Source and dilution of primary antibodies

	Source	Dilution (application)
AKT	Abcam#ab131443	1:10,000 (WB)
		1:100 (IF)
p27	Abcam#ab32034	1:1000 (WB)
CRALBP	Abcam#ab15051	1:1000 (IF)
		1:1000 (WB)
S-100	Abcam#ab4066	1:200 (WB)
PTEN	Abcam#ab32199	1:10,000 (IF)
		1:10,000 (WB)
cyclinA2	Abcam#ab32386	1:10,000 (WB)
p21	Abcam#ab109520	1:2000 (WB)
		1:1000 (IF)
PAR2	Abcam#ab180953	1:2000 (WB)
cyclinB1	Abcam#ab181593	1:2000 (WB)
		1:500 (IF)
PDK2	Novusbio#NBPI-87307	1:200 (WB)
KLF4	Abcam#ab215036	1:10,000 (WB)
OCT4	Abcam#ab18976	1:100 (IF)
RPE65	Abcam#ab13826	1:5000 (WB)
cyclinD1	Abcam#ab134175	1:10,000 (WB)
		1:50 (IF)
β-actin	SIGMA#A5441	1:3000 (WB)

### Immunofluorescence assay

The immunofluorescence assay was performed according to the previously described methods [30]. The primary antibodies used were shown in Table 2. The secondary antibodies used were Alexa 488 goat-anti mouse lgG (1:1000, Invitrogen) and Alexa Fluor 594 donkey anti-rabbit IgG (1:1000, Invitrogen). The cells were analyzed under an LSM780 or LSM800 confocal microscope (Zeiss, Germany).

### Statistical analyses

All values were presented as the means ± standard deviation (SD). The statistical analyses were performed with GraphPad Prism software. A 2-tailed unpaired Student’s *t* test was used for analyses comparing 2 groups. *P* values < 0.05 were considered significant.

## Results

### Phenotype of hRPE cells and mouse ESCs

**Fig. 1:**
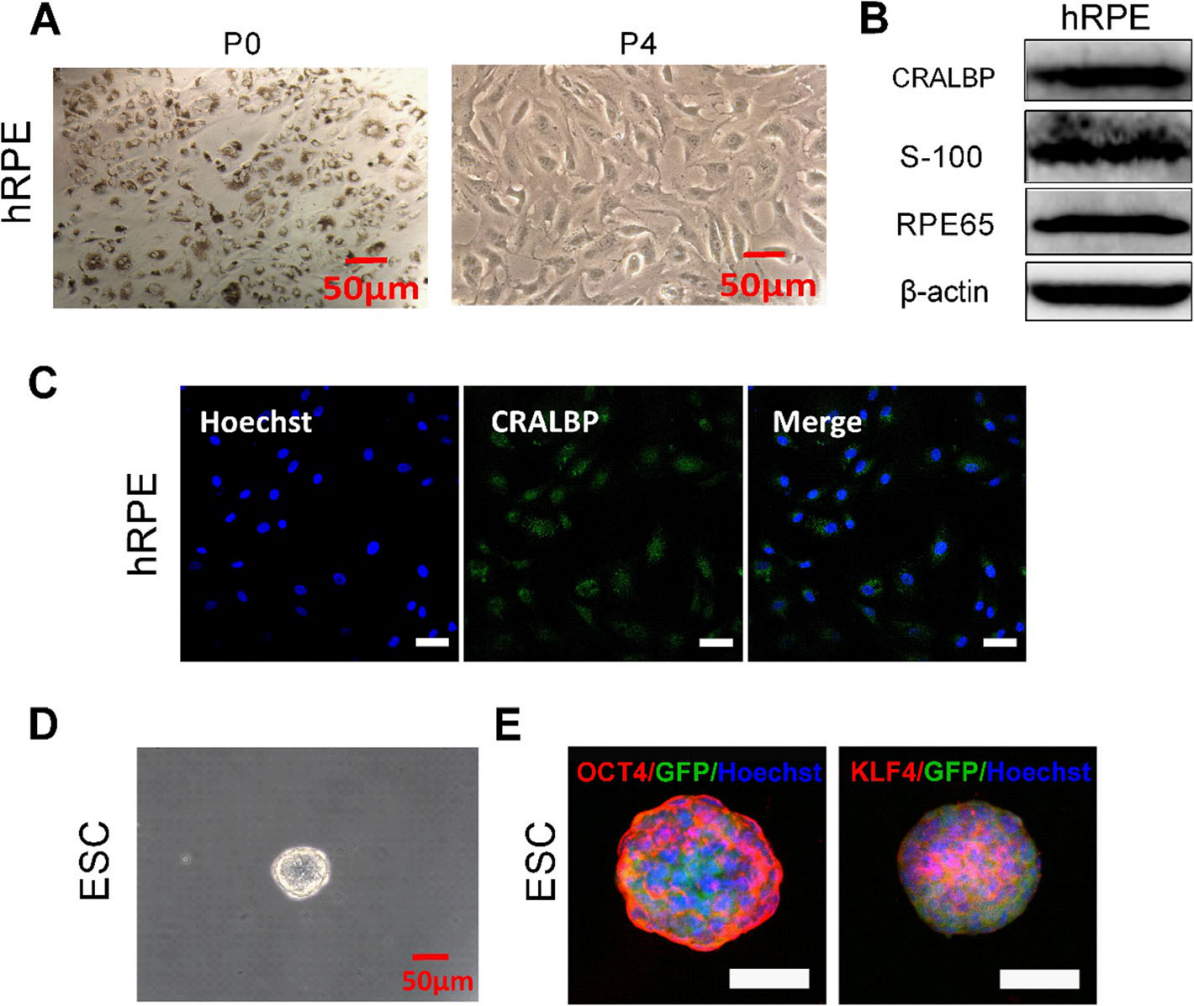
Characteristics of hRPE cells and ESCs. **a** Representative images of hRPE cells by phase microscopy. **b** Western blotting of CRALBP, S-100, and RPE65 in hRPE cells. β-actin served as the internal control. **c** Immunofluorescence assays of CRALBP in hRPE cells. Scale bar, 50 μm. **d** Representative images of ESCs by phase microscopy. **e** Immunofluorescence assays of OCT4 and KLF4 in ESCs. Scale bar, 50 μm

The primary adherent pigmented hRPE cells at first plating (P0) reached 90% confluence after 7 days of cultivation (Fig. 1a). They grew as cobblestone cultures and contained a great quantity of pigmentation. During culture, the pigment was diluted upon cell division and the cells gradually acquired a fusiform, largely depigmented morphology. The results of western blotting indicated that the differentiation marker proteins CRALBP, S-100, and RPE65 were expressed in the hRPE cells at P4 (Fig. 1b). Immunofluorescence staining also showed CRALBP expression in the hRPE cells (Fig. 1c).

The mouse ESCs exhibited a clonal or islet appearance. Under a light microscope, the clone was bright and round with a clear, sharp boundary (Fig. 1d). Immunofluorescence assays showed that the stem cell markers OCT4 and KLF4 were expressed in the ESCs (Fig. 1e).

### Effects of coculture with ESCs on morphological changes in hRPE cells

**Fig. 2:**
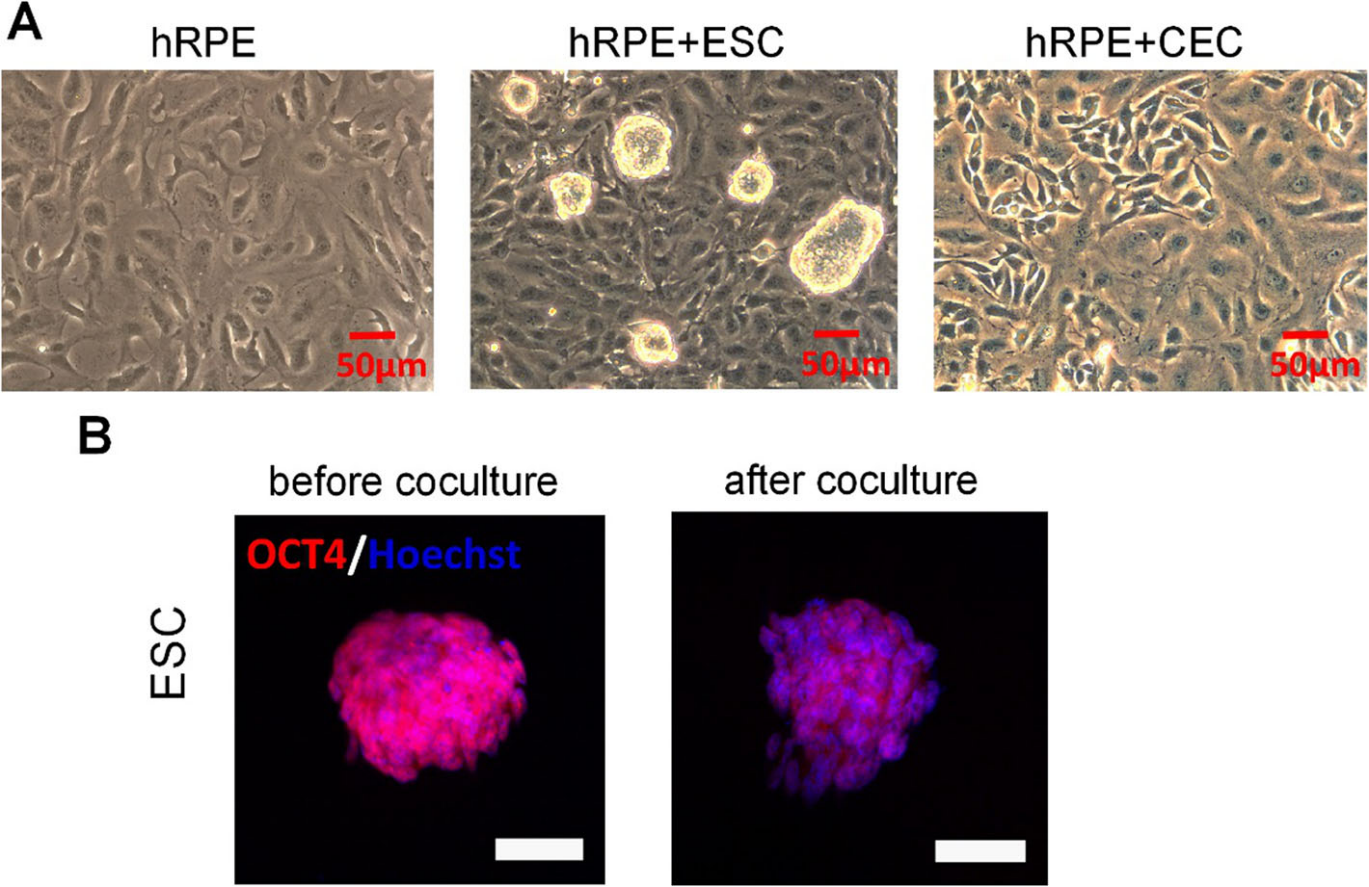
Effects of coculture with ESCs on morphological changes in hRPE cells. **a** Representative images of morphology by phase microscopy. **b** The expression of OCT4 in ESC before and after coculture as determined by immunofluorescent staining. Scale bar, 50 μm

The role of ESCs in regulating the morphology of hRPE cells was investigated. The hRPE cells at P5 in the control group presented a fusiform pattern (Fig. 2a). On the other hand, the P5 hRPE cells in the hRPE+ESC group showed an epithelioid shape with a homogeneous morphology that is more similar to the primary cultured cells originating from the eyecups, which maintained a normal cell property of contact inhibition. Immunofluorescent staining indicated reduced OCT4 expression in ESCs cocultured with hRPE cells (Fig. 2b). After culturing for 72 h, hRPE cells from all groups were collected for experiments below.

### Coculture with ESCs enhances the proliferative capacity of hRPE cells

**Fig. 3:**
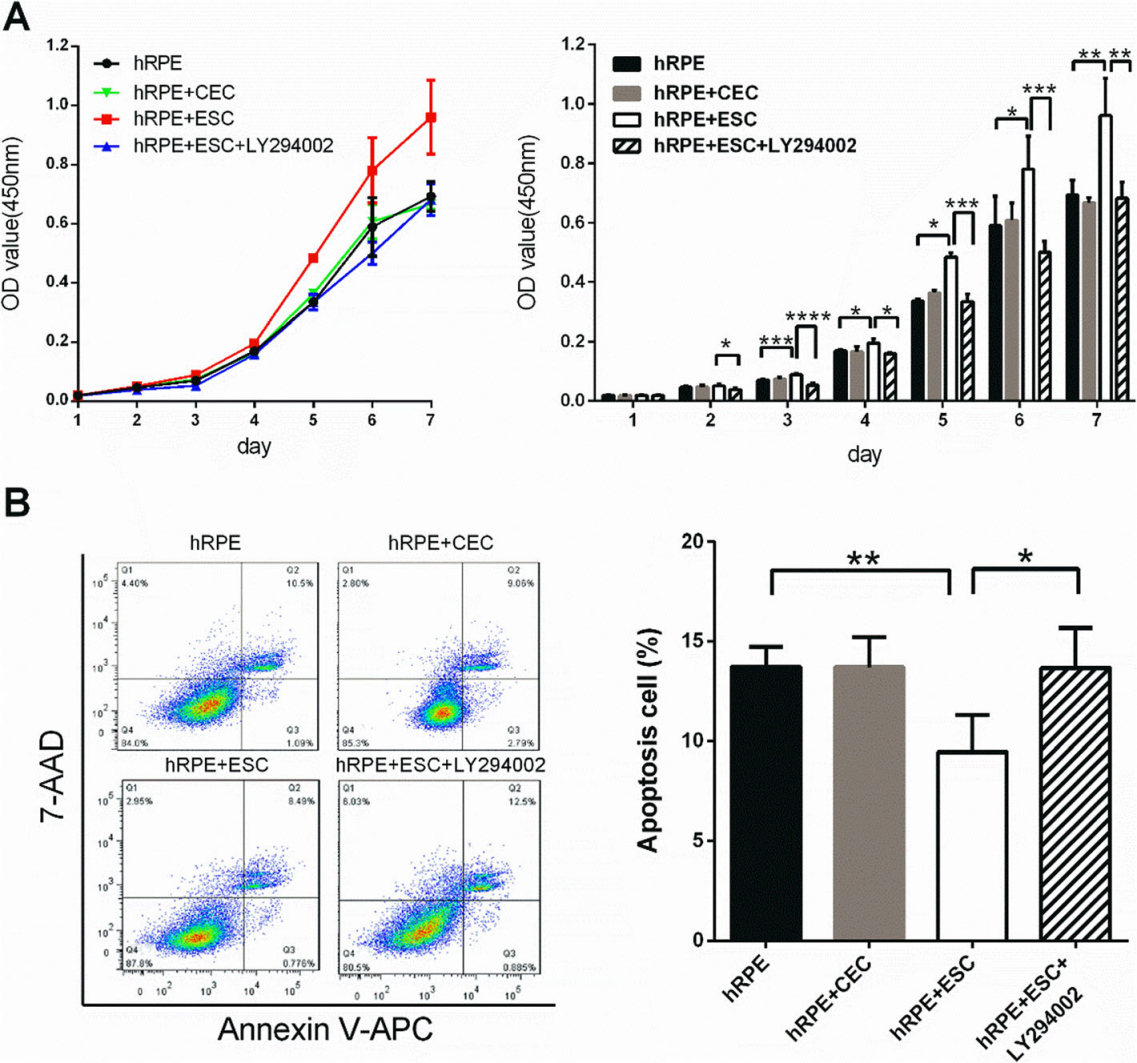
Coculture with ESCs increases the proliferation rate and lowers the apoptosis rate. **a** Proliferation of hRPE cells sorted from the control (hRPE), hRPE+CEC, hRPE+ESC, and hRPE+ESC+LY294002 groups, as assessed by a CCK8 proliferation assay. **b** Percentages of apoptotic hRPE cells, as assessed by flow cytometer. Data are means ± SDs. *P < 0.05; **P < 0.01; ***P < 0.001; ****P < 0.0001

We next investigated the potential effects of ESCs on the proliferation of hRPE cells. The CCK-8 test (a common assay for detecting cell proliferation) was used to obtain the growth curve of hRPE cells from each group. During the slow-growing latent phase in days 1 and 2, no marked differences of optical density (OD) values were detected among the three groups (Fig. 3a). However, on the third day, the hRPE+ESC group showed significantly higher OD values than the control group. All three groups entered the logarithmic growth phase on the fourth day but the slope of the growth curve for the hRPE+ESC group was higher than the other two groups. These observations indicate that the hRPE cells treated with ESCs possessed a relatively stronger growth capacity. In contrast, there were no significant differences in the growth curves between the hRPE group and the hRPE+CEC group.

To study the effects of ESCs on cell apoptosis, we employed Annexin V-APC/7-AAD staining and flow cytometry analysis. We found that the percentages of apoptotic hRPE cells from the Ctrl, hRPE+CEC, and hRPE+ESC groups were 13.73 ± 0.9912%, 13.7 ± 1.512%, and 9.473 ± 1.835%, respectively (Fig. 3b). There were fewer apoptotic cells in the hRPE+ESC group than in the hRPE group (*P* = 0.0065), while the percentages of apoptotic cells were almost the same in the hRPE and hRPE+CEC groups. These results suggest that ESCs inhibit apoptosis in hRPE cells.

**Fig. 4:**
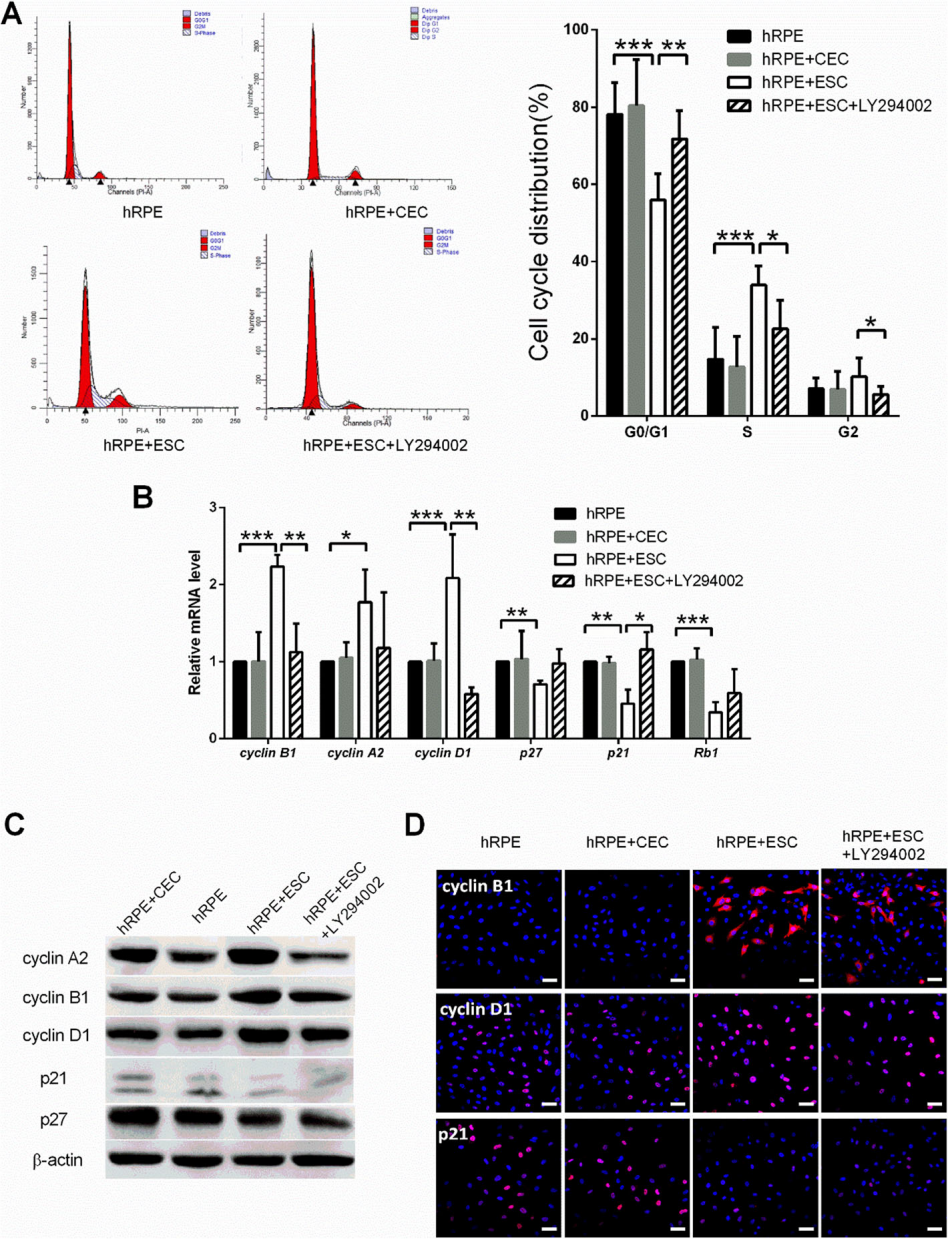
ESC-treated hRPE cells had a higher proliferation rate with a faster cell cycle turnaround time. **a** Proportion of cell cycle distribution in C918 cells, as assessed by flow cytometry. **b** Expression of the cell cycle related factors in hRPE cells, as assessed by RT-qPCR. **c** Western blotting of cyclin proteins and p21, p27 in hRPE cells. β-actin served as the internal control. **d** The expression of cyclin B1, cyclin D1, and p21 in hRPE cells as determined by immunofluorescent staining. Scale bar, 50 μm. Data are means ± SDs. *P < 0.05; **P < 0.01; ***P < 0.001

Cell cycle progression was further evaluated by flow cytometry. As shown in Fig. 4a, the percentage of hRPE cells entering the cell cycle was significantly higher in the hRPE+ESC group than the other groups (*P* < 0.05). In the hRPE+ESC group, 33.94% ± 2.191% of cells were entering S phase, whereas in the hRPE group, 14.71% ± 2.468% of cells were in S phase. Consistent with the flow cytometry results, hRPE cells cocultured with ESCs showed higher expression levels of the cell cycle promoters cyclin A2, cyclin B1, and cyclin D1, and lower expression levels of the cell cycle negative regulators p21 and p27, both transcriptionally and translationally (Fig. 4b–d). In contrast, neither the cell cycle distribution nor the cell cycle-related protein expression levels of hRPE cells were significantly changed by coculture with CECs.

### Coculture with ESCs enhances the stem cell phenotype of hRPE cells

**Fig. 5:**
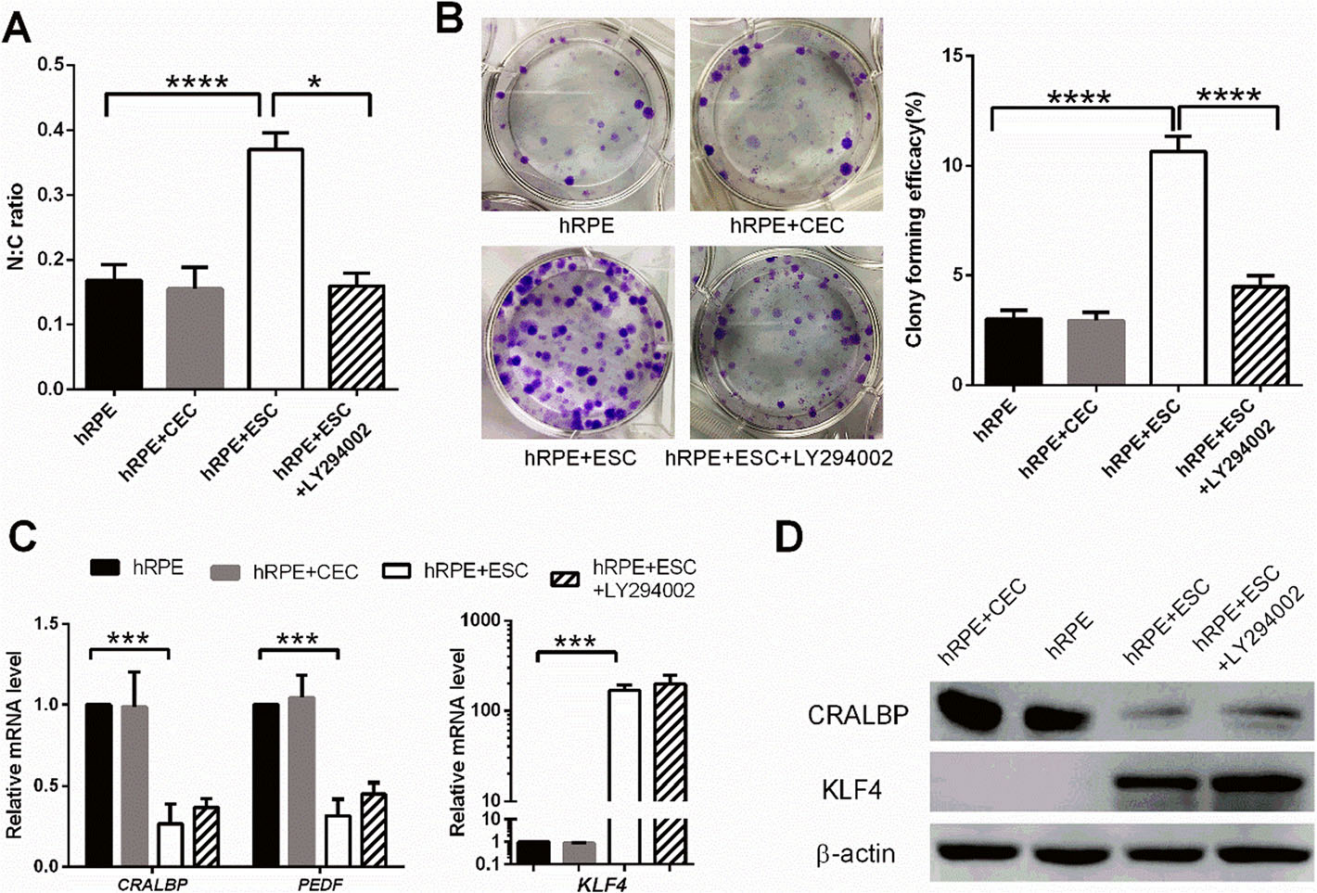
Coculture with ESCs enhances the stem cell phenotype of hRPE cells. **a** The N:C ratio analysis of hRPE cells. **b** Colony-forming efficacy and representative images of clones formed by hRPE cells. **c** Expression of the RPE differentiation markers (CRALBP and PEDF) and stem cell-associated marker (KLF4) in hRPE cells, as assessed by RT-qPCR. **c** Western blotting of CRALBP and KLF4 in hRPE cells. β-actin served as the internal control. Data are means ± SDs. *P < 0.05; ***P < 0.001; ****P < 0.0001

As shown in Fig. 2a, the hRPE cells cocultured with ESCs had smaller cell sizes with bigger nucleus to cytoplasm (N:C) ratios than cells in the control group (Fig. 5a). No obvious differences in morphology and N:C ratio was observed between the control and hRPE+CEC groups. To evaluate the clonal growth capacity, the hRPE cells from the three groups were seeded without a feeder layer to assess colony-forming efficiency (CFE). At day 7, the CFE of the hRPEs in the ESC-treated group reached 10.65 ± 0.6856%, whereas the CFE of the control and hRPE+CEC groups were 3 ± 0.4082% and 2.925 ± 0.3775%, respectively (Fig. 5b).

RT-qPCR analysis revealed that the mRNA expression of the RPE differentiation markers CRALBP and PEDF by hRPE cells in the hRPE+ESC group was significantly decreased by 0.264- and 0.315-fold, respectively, in comparison to the control group (Fig. 5c). However, the expression of KLF4, a marker associated with early stem cells and reprogramming, was markedly increased by 167-fold in hRPE cells after coculturing with ESCs (Fig. 5c). Consistent with the mRNA expression profiles, western blot analysis revealed that hRPE cells from the control group strongly expressed CRALBP, while hRPE cells in the hRPE+ESC group showed weaker CRALBP expression (Fig. 5d). KLF4 was barely detectable in the hRPE cells in the control group but markedly upregulated in ESC-treated hRPE cells (Fig. 5d). In contrast, the expression of RPE-specific and stem cell-associated markers in hRPEs from the hRPE+CEC group was not significantly different from that of the control group. These observations indicate that ESCs can enhance the stem cell phenotype of hRPE cells.

### ESCs enhance the proliferative capacity of hRPE cells by activating the PI3K pathway

**Fig. 6:**
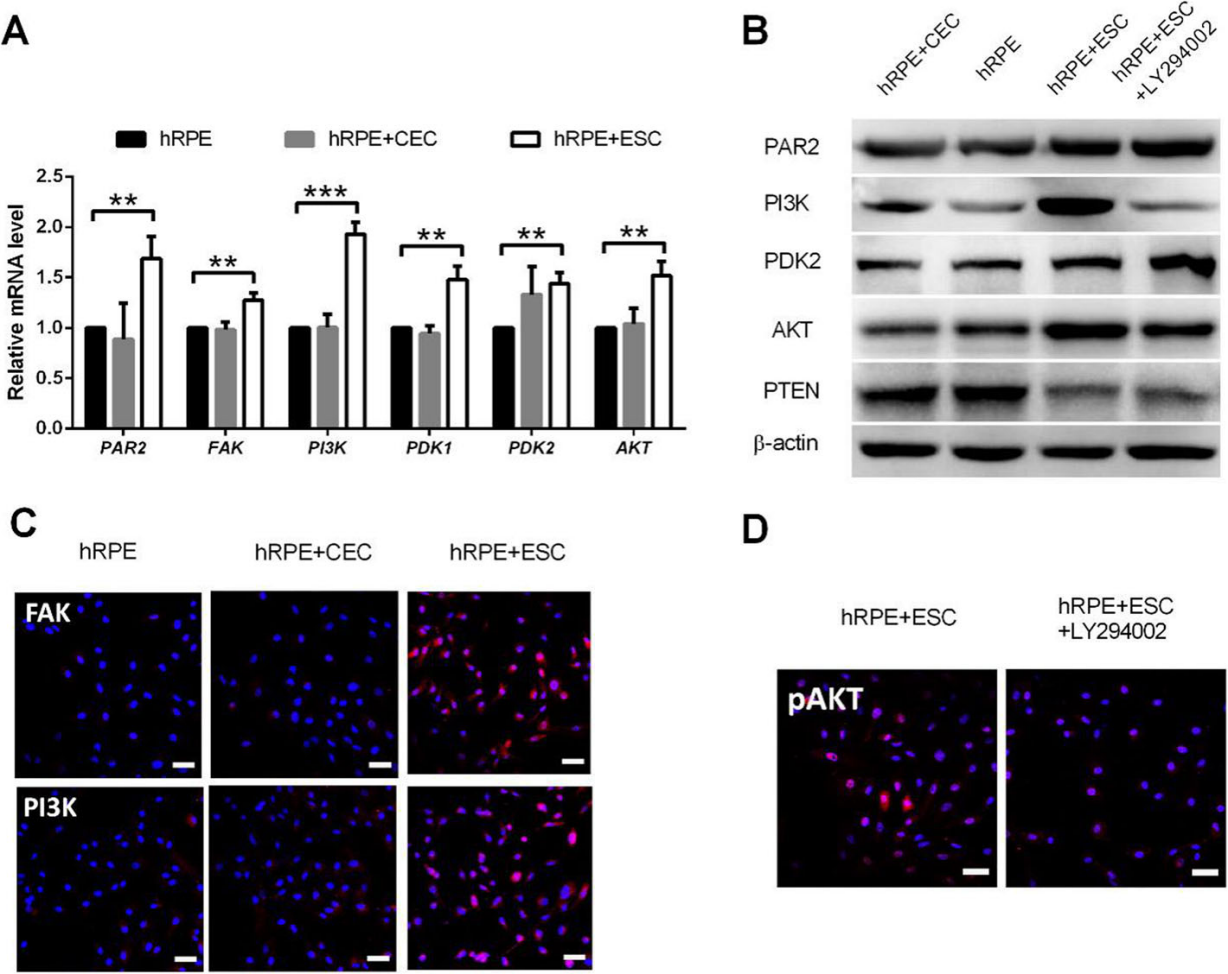
ESCs enhance the proliferative capacity of hRPE cells by activating the PI3K pathway. **a** Expression of PI3K pathway genes in hRPE cells, as assessed by RT-qPCR. **b** Western blotting of PI3K pathway genes in hRPE cells. β-actin served as the internal control. **c** The expression of FAK and P3K in hRPE cells as determined by immunofluorescent staining. Scale bar, 50 μm. **d** Immunofluorescence assays of pAKT in hRPE cells. Scale bar, 50 μm. Data are means ± SDs. **P < 0.01; ***P < 0.001

Our previous finding [27] demonstrated that the ESC microenvironment promoted the proliferation of corneal epithelial cells via activation of the PI3K/Akt signaling pathway. Therefore, we further examined whether coculturing with ESCs enhances the proliferative capacity of hRPE cells by upregulating the PI3K pathway. We performed RT-PCR validation of key PI3K pathway genes, including *PAR2*, *FAK*, *PI3K*, *PDK1/2*, and *AKT*, and found that they were significantly upregulated in hRPE cells after exposure to ESCs (Fig. 6a–c). In contrast, the expressions of these genes were not enhanced in hRPE cells from the CEC group compared with the hRPE group.

To determine whether PI3K pathway activity is necessary for the pro-proliferative effect of the ESC microenvironment, we added the PI3K antagonist, LY294002 to disrupt PI3K signaling (Fig. 6d). LY294002 abolished the pro-proliferative effect of the ESCs on hRPE cells in the ESC co-culture system; the growth of hRPE cells was reduced and ESC-inhibited apoptosis was blocked (Figs. 3 and 4). Meanwhile, expression of the cell cycle promoters cyclin A2, cyclin B1, and cyclin D1 decreased, while the expression of p21 and p27 increased significantly (Fig. 4b–d). These findings indicate that the PI3K pathway is indeed activated by ESCs in hRPE cells, which in turn boosts proliferation. Interestingly, although LY294002 decreased the N:C ratio and clonal growth capacity of hRPEs (Fig. 5a, b), it did not affect their expression of RPE-specific and stem cell-associated markers (Fig. 5c, d).

## Discussion

A variety of signaling pathways, such as the MAPK, PI3K, and Notch pathways, become deficient or inactive in senescent cells, including senescent adult stem cells, leading to anti-proliferation of the organ’s stem cells, which then prevents their regeneration [33–36]. With the rapid development in bioengineering, in vivo applications of biopolymers that release signaling pathway agonists could evoke efficient regenerative responses from senescent cells [37–39]. However, achieving “young” levels of signaling not only promotes cell growth, but also cause undesired changes, including but not limited to oncogenic transformation [40–42]. Additionally, even young cells are doomed to perish when introduced into an old organ without a youthful niche [43, 44]. Though the recent study demonstrated that sub-tenon WJ-MSCs administration was effective on reactivating the degenerated photoreceptors in dormant phase [13], the long-term effects may be weakened with the gradual aging of WJ-MSCs in vivo, and the senescence-associated secretory phenotype secreted by senescent WJ-MSCs may even produce adverse effects. Therefore, the idea of providing endogenous or transplanted stem and progenitor cells with better microenvironments is being pursued by many researchers in the field of regenerative medicine.

We and others have demonstrated that the ESC microenvironment manifests a pro-regenerative activity that enhances and, more importantly, rejuvenates the regenerative capacity and promotes the proliferation of differentiated cells. Differentiated ESCs and tissue-specific adult stem cells yield much weaker results than ESCs [21, 24, 43]. Administration of sub-tenon WJ-MSCs could stop the progression of retinal degeneration and rescue photoreceptors in the dormant phase with the paracrine effects of WJ-MSCs [13]. Interactions between cells involve multi-directional signaling. In the coculture system, the stem cell microenvironment serves as a niche by releasing cytokines via paracrine and autocrine pathways as well as direct signal transmission via cell-to-cell contact. We have previously shown that the enhancement of the stem cell phenotype and proliferation of CECs by ESCs was much more remarkable in the cell-to-cell contact coculture system than in the non-contact system which only exists the paracrine effect. Furthermore, CECs with strong proliferation after being cocultured with ESCs were not reprogrammed to pluripotency and were not tumorigenic, suggesting that the ESC microenvironment could safely and effectively improve the proliferative potential of adult cells [26]. Accordingly, we cultured hRPE cells alone or with ESCs in a cell-to-cell contact coculture system. Compared with the control group, ESC-treated hRPE cells had a higher proliferation rate with a faster cell cycle turnaround time, a decreased apoptotic rate, an increased clone formation rate, and an increased stemness. However, CECs do not share the ability of ESCs to influence the regenerative behavior of hRPE cells, which support the notion that non-embryonic cells cannot promote the proliferative ability of terminal cells.

With increased aging, RPE cells show a reduced activation of the PI3K/Akt pathway and are less tolerant to tissue damage [45]. Studies have shown that epidermal growth factor and thrombin can stimulate RPE cell survival and proliferation through activating the PI3K pathway [46, 47]. Our previous studies demonstrated that the proliferation of corneal epithelial cells was significantly promoted by the ESC microenvironment via activation of the PI3K/Akt signaling pathway [26, 29]. Consistent with these findings, the present study showed that ESCs upregulate the PI3K pathway of hRPE cells, which accounts for their pro-proliferative effect.

An accumulation of evidence supports a key role for the PI3K pathway in cell cycle progression. Transcription and translation of multiple cyclins have been shown to be dependent on the activation of the PI3K/Akt pathway [48]. Cyclin D1 plays a central role in cell cycle progression from G1 to S phase [49], and its accumulation induces RPE cell proliferation [47]. Cyclin A is involved in DNA replication in S phase and is required for G2/M phase transition [50]. Cyclin B facilitates G2/M progression; depletion of cyclin B causes a dramatic G2 arrest and reduction in mitotic cells in hTert-RPE1 cells [51]. Moreover, PI3K activation reduces the levels of cyclin-dependent kinase inhibitors (CDKIs). Both p21 and p27 are important CDKIs with a wide range of kinase inhibition activity that can effectively block the activity of cyclin/CDK complexes to prevent cells from going through the G1/S phase checkpoint and inhibit cell proliferation [52]. It was revealed that the level of p21 increases in an age-dependent manner; hence, p21 has been used as a senescence marker [53–55]. It has also been demonstrated that increased p21 expression in RPE cells inhibits their proliferation in vitro [56]. Consistent with these findings, our experiments showed that ESCs activate the PI3K pathway of hRPE cells, which accounts for their pro-proliferation effect via upregulating cyclins and downregulating CDKIs.

Tissue stem cells have been found in tissues with lower self-renewal demands such as the nervous system, and where a rapid rate of cellular turnover is required, such as the skin [57, 58]. Adult stem cells are an important cell source in regenerative medicine. Normally, cell differentiation starts from stem cells that progress to transiently amplifying cells (TACs) and finally to terminally differentiated cells. This process can be delayed or even reversed by certain factors or conditions [59]. It has been shown that a sub-population of multipotent, self-renewing RPESCs is present in the human RPE layer [17]. Although there are no definitive markers to identify RPESCs, RPESCs show higher expression of c-MYC and KLF4 and lower expression of CRALBP, Tyr, and PEDF compared to RPE cells [17, 60]. A combination of stem cell-associated and differentiation markers has enabled scientists to identify putative RPESCs. The present study found that the isolated hRPE cells from the coculture systems expressed a significantly higher level of the key stemness factor KLF4 at the protein and transcription levels and decreased level of the differentiation marker CRALBP. Our results suggest that ESCs, through coculture, can enhance the stem/progenitor phenotype of hRPE cells. As KLF4 is an upstream regulator of the PI3K/Akt pathway [61], the activation of KLF4 in hRPE cells by ESCs cannot be affected by PI3K inhibitors.

## Conclusions

Our study provides evidence that the ESC microenvironment can enhance the undifferentiated status and proliferation properties of hRPE cells. This finding opens new avenues for the potential therapeutic application of ESCs in regenerative medicine. Further studies on the functional enhancement of hRPE cells in the coculture system may shed light on the use of such cells in regenerative medicine.

## Data Availability

Most data generated or analyzed during this study are included in the article. Additional data sets generated and/or analyzed during the study and other relevant information are available from the corresponding authors upon reasonable request.

## Abbreviations

AMD: Age-related macular degeneration
RPE: Retinal pigment epithelium
ESC: Embryonic stem cell
iPSCs: Induced pluripotent stem cells
RPESCs: Retinal pigment epithelium stem cells
CEC: corneal epithelial cell
CCK8: Cell Counting Kit-8
DiI: 1,1′-Dioctadecyl-3,3,3′,3′-tetramethylindocarbocyanine perchlorate
DiD: 1,1-Dioctadecyl-3,3,3,3-tetramethylindodicarbocyanine
PI3K: Phosphoinositide 3-kinase
PDK2: Pyruvate dehydrogenase kinase isoform 2
mTOR: Mechanistic target of rapamycin
MSCs: Mesenchymal stem cells
RT-qPCR: Reverse transcription polymerase chain reaction
KLF4: Kruppel-like factor 4
SASP: Senescence-associated secretory phenotype
PAR2: Protease-activated receptor 2
CRALBP: Cellular retinaldehyde-binding protein
OCT4: Octamer-binding transcription factor 4
PTEN: Phosphatase and tensin homologue deleted on chromosome ten
FAK: Focal adhesion kinase
